# Comparative analysis of subsampling methods for large mosquito samples

**DOI:** 10.1186/s13071-019-3606-5

**Published:** 2019-07-16

**Authors:** Linda Jaworski, Stephanie Jansen, Wolf Peter Pfitzner, Matthias Beck, Norbert Becker, Jonas Schmidt-Chanasit, Ellen Kiel, Renke Lühken

**Affiliations:** 1 Carl von Ossietzky University, Oldenburg, Germany; 2 Bernhard Nocht Institute for Tropical Medicine, WHO Collaborating Centre for Arbovirus and Hemorrhagic Fever Reference and Research, Hamburg, Germany; 3 German Mosquito Control Association (KABS), Waldsee, Germany; 4 Centre for Infection Research (DZIF), Partner Site Hamburg-Lübeck-Borstel-Riems, Hamburg, Germany

**Keywords:** Mosquito surveillance, Large mosquito samples, Subsampling

## Abstract

**Background:**

The analysis of large mosquito samples is expensive and time-consuming, delaying the efficient timing of vector control measurements. Processing a fraction of a sample using a subsampling method can significantly reduce the processing effort. However, a comprehensive evaluation of the reliability of different subsampling methods is missing.

**Methods:**

A total of 23 large mosquito samples (397–4713 specimens per sample) were compared in order to evaluate five subsampling methods for the estimation of the number of specimens and species: area, volume, weight, selection of 200 random specimens and analyses with an image processing software. Each sample was distributed over a grid paper (21.0 × 29.7 cm; 25 grid cells of 4.2 × 5.9 cm) with 200 randomly distributed points. After taking pictures, mosquito specimens closest to each of the 200 points on the paper were selected. All mosquitoes per grid cell were identified by morphology and transferred to scaled tubes to estimate the volume. Finally, the fresh and dry weights were determined.

**Results:**

The estimated number of specimens and species did not differ between the area-, volume- and weight-based method. Subsampling 20% of the sample gave an error rate of approximately 12% for the number of specimens, 6% for the proportion of the most abundant species and between 6–40% for the number of species per sample. The error for the estimated number of specimens using the picture processing software ImageJ gave a similar error rate when analyzing 15–20% of the total sample. By using 200 randomly selected specimens it was possible to give a precise estimation of the proportion of the most abundant species (*r* = 0.97, *P* < 0.001), but the number of species per sample was underestimated by 28% on average. Selecting adjacent grid cells instead of sampling randomly chosen grid cells and using dry weight instead of wet weight did not increase the accuracy of estimates.

**Conclusions:**

Different subsampling methods have various advantages and disadvantages. However, the area-based analysis of 20% of the sample is probably the most suitable approach for most kinds of mosquito studies, giving sufficiently precise estimations of the number of specimens and species, which is slightly less laborious compared to the other methods tested.

**Electronic supplementary material:**

The online version of this article (10.1186/s13071-019-3606-5) contains supplementary material, which is available to authorized users.

## Background

Globalization and climate change resulted in the worldwide spread of invasive mosquito species and associated pathogens including arthropod-borne viruses (arboviruses), nematodes and protozoans [1]. For example, the establishment of the exotic Asian tiger mosquito (*Aedes albopictus*) in Europe caused five outbreaks of the exotic chikungunya virus in France and Italy during the last 10 years with at least 605 human cases [2–8]. Therefore, surveillance programs are implemented in many countries worldwide in order to detect the circulation of native and exotic pathogens or to identify changes in mosquito species compositions.

Most surveillance programs use baited mosquito traps (e.g. light and/or carbon dioxide), allowing mass trapping of several thousand or more specimens per trapping night [9]. These data provide information about the abundance and species composition of mosquitoes in the studied areas, which is a basic prerequisite to understand pathogen circulation or to perform effective control measurements like spatial-temporal application of larvicides or adulticides [10]. However, the identification of all specimens in large samples can be time consuming and therefore can be quite expensive. Faster sample processing for example can allow a more efficient timing of vector control measurements. Subsampling, i.e. analyses of a fraction of the sample and subsequent extrapolation, can be a suitable strategy to reduce the effort of sample analysis. Thereby, an optimal subsampling method should save resources, but still give reliable estimates of the number of mosquito specimens and species per sample.

Subsampling of invertebrate samples is a common method in different fields of ecology, e.g. for samples of macroinvertebrates [11] or parasites [12, 13]. Common methods for adult mosquito samples are random subsampling based on area [14, 15], volume [16], weight [17] or random selection of a fixed number of specimens (e.g. 200 mosquitoes) [18–21]. Some studies also combined different methods, e.g. random subsampling of specimens in combination with an extrapolation per weight [18, 22–32] or specimens selected by area and extrapolated by weight [33].

However, only a few studies compared the precision of the applied estimation method. A comparative study was conducted by Van Ark [34], investigating the reliability of subsampling based on volume and weight of large light trap catches including mosquitoes. This study revealed a more reliable estimation based on the weight compared to the volume. Debevec [17] used a weight-based subsampling method and found a positive linear correlation between the abundance per subsample and the total number of specimens. In addition, a subsample of at least 30% was identified suitable to estimate the species richness. Another study determined a positive correlation between the number of specimens in a random subsample of 30 specimens and the total number of specimens for a common species [19]. Burkett-Cadena et al. [18] observed a positive correlation between the estimated and actual counts of different species, using a combination of random subsampling with an extrapolation per weight. Furthermore, Kesavaraju and Dickson [35] tested a quick technique to estimate the number of mosquito specimens from standardized pictures analyzed with an image processing software. Optimized calibration facilitates a reliable estimation of the number of specimens.

Although different subsampling methods are used in mosquito studies, a comprehensive evaluation of different estimation methods is missing. The reliability of the applied estimation method has direct implications for the interpretation of mosquito monitoring results. Therefore, the objective of this study was to compare five different, commonly applied techniques (subsampling by area, volume or weight, selection of random specimens and analyses of pictures from the samples) to estimate the number of adult mosquito specimens and species. The study presents a systematic comparison of all five methods and discusses the potential applicability regarding their estimation accuracy and time efficiency.

## Methods

A total of 23 samples of adult mosquitoes were collected on four dates between 7 June 2016 and 21 July 2016 within a monitoring program of the German Mosquito Control Association in 12 trapping stations along the floodplains of the Upper Rhine Valley. Mosquitoes were sampled with Heavy Duty Encephalitis Vector Survey traps (EVS trap, BioQuip Products, Rancho Dominguez, CA, USA) baited with 1.5 kg of dry ice. Samples were stored at −20 °C until processing. These samples compromised a total of 37,557 mosquitoes, with an average (±SD) of 1632.2 ± 1135.1 specimens and 8 ± 1.4 species per sample (Additional file 1: Table S1, Additional file 2: Table S2).

**Fig. 1:**
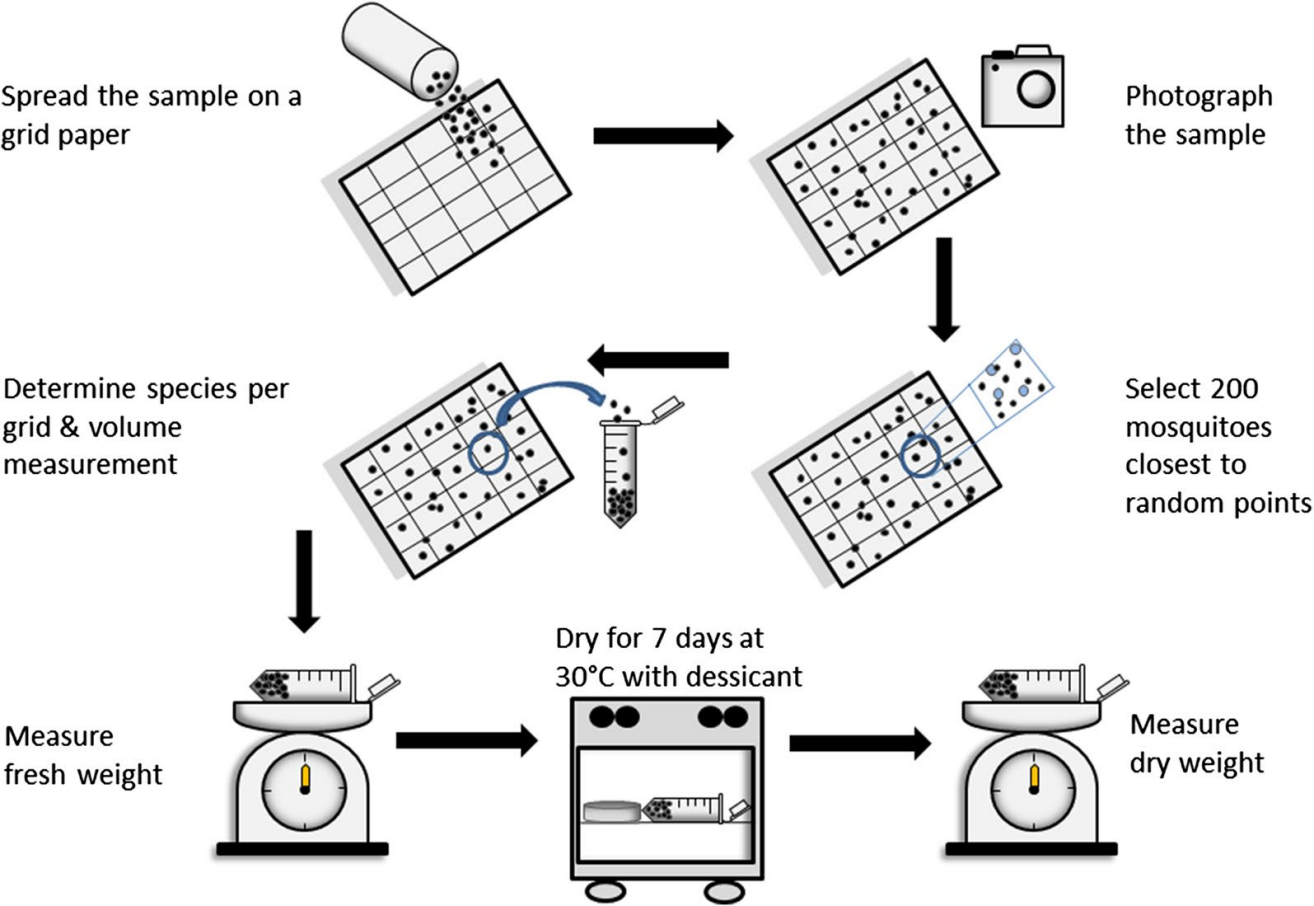
Workflow of the sample processing

Five different subsampling methods to estimate the number of mosquito specimens and species per sample were compared: extrapolation by volume, area, and weight, image processing (number of specimens only) and random selection of 200 specimens (number of species only) (Fig. 1). The same workflow was applied for each sample. Mosquitoes were uniformly distributed over a sheet of paper (21.0 × 29.7 cm subdivided into 25 grid cells, 4.2 × 5.9 cm per cell) with 200 randomly distributed blue points; Additional file 3: Figure S1). Non-mosquito invertebrates and plant materials (e.g. leaves or wood waste) were removed. The paper with the sample was placed on a laboratory bench with light from a 100-W neon bulb. Clustered accumulations of mosquito specimens were avoided by re-sorting the sample. Photos were taken at a vertical distance of approximately 120 cm with a camera pointing straight downwards (Olympus OMD EM5, Olympus, Shinjuku, Tokyo, Japan). Thereby, we deliberately refrained from using special equipment to test the approach under field conditions, e.g. no photo developing tray or tripod were used [36]. Every sample was photographed three times to estimate the reproducibility of this method. The mosquitoes were rearranged between the images to alter the distribution pattern of specimens. Next, the mosquito specimen closest to each of the 200 random points on the paper was selected. The corresponding grid cell numbers of each of these specimens were recorded. Subsequently all mosquitoes per grid cell were identified based on morphology [36]. Depending on the size of the sample, mosquito specimens of each grid cell were stored in 2 ml (Eppendorf, Hamburg, Germany) or 15 ml tubes (Sarstedt, Nümbrecht, Germany). The measurement of the volume per sample was conducted by first tapping the tube for 10–15 times on a table to concentrate the sample on the bottom of each container. The volume per subsample was estimated from the volume scale on each tube. Finally, weight measurements were conducted for each subsample in the same tubes used for the volume measurement. The fresh weight was determined by weighing each tube using an electronic scale (Sartorius R160P electronic semi-microbalance, Sartorius, Göttingen, Germany). For the dry weight analysis, tubes were kept open in an oven (Memmert type 400, Memmert, Schwabach, Germany) for seven days at 30 °C. A mix of rice and salt was added to bind the moisture as a cheap and easily accessible desiccant under field conditions. The drying substance was exchanged every day. Finally, the empty weight of each tube was determined to calculate the fresh and dry weight for each subsample.

### Statistical analysis

The subsampling data were analyzed with R [37] using the packages *magrittr* [38], *plyr* [39] and *tidyr* [40]. Results were visualized with *ggplot2* [41] and *cowplot* [42]. A bootstrap approach was applied to estimate the accuracy for the estimation of the number of mosquito specimens and species in relation to the proportion of each sample analyzed. As a basis for the analysis of the different subsampling methods, 1–25 raster cells were randomly selected 1000 times without replacement for each sample. The average number of specimens per cell was calculated and multiplied by the total number of cells (*n* = 25) for the area-based approach. Alternatively, the volume or dry/fresh weight of each subsample relative to the volume or weight of total sample was used to estimate the total number of specimens. The correlation between the dry and fresh weight was evaluated with paired samples t-test. All these estimates were divided by the actual number of specimens per sample, giving a consistency score of over- or underestimation, i.e. estimated number/actual number ×100. For each number of selected cells (1–25), the bootstrap means and confidence intervals (95%) of the consistency scores were calculated and averaged over the 23 samples. In addition, to evaluate the relevance of a random selection of grid cells, different proportions of adjacent grid cells (20, 40, 60 or 80%) were selected in a single step (Additional file 4: Figure S2). Mean and standard deviation of the percentage of detected specimens were calculated over all samples. Using the same bootstrapping method, the proportion of the most abundant species and the number of detected species per sample were evaluated. There is only one most abundant species in each sample, but this could be a different species per sample. As the for the total number of specimens and species, this value is calculated relative to the actual value. Thus, the data can be analysed together.

The number of species and proportion of the most abundant species based on 200 randomly selected specimens was compared to the actual values for each sample. The mean and standard deviation of the percentage of detected species were calculated over all samples. Pearson’s product-moment correlation was used to analyze the statistical relationship between the number of detected species and proportion of the most abundant species in the randomly selected 200 specimens and the actual sample.

Finally, the open-source image processing software ImageJ [43] was used to evaluate the number of specimens per sample. The processing of the images was performed according to Kesavaraju and Dickson [35]. The estimate of the total number of specimens predominantly depends on two variables, THRESHOLD (differentiation of mosquitoes from background) and SIZE (minimum area classified as an object). To identify the combination of both variables giving the best estimate, a macro for each combination of both variables THRESHOLD (1–100, in steps of 1) and SIZE (1–100, in steps of 1) was run for each sample using an automatic script (Additional file 5: Text S1). Again, the consistency of the estimation compared to the actual number of specimens was evaluated by comparison with the actual number of specimens per sample, while the best combination was identified by a mean consistency between 97.5% and 102.5% with a minimal standard deviation over all samples tested. In addition, the reproducibility of the measurement was tested for the three replicated pictures per sample. The statistical relationship between the number of estimated and detected number of specimens per sample was analyzed with Pearson’s product-moment correlation.

## Results

**Fig. 2:**
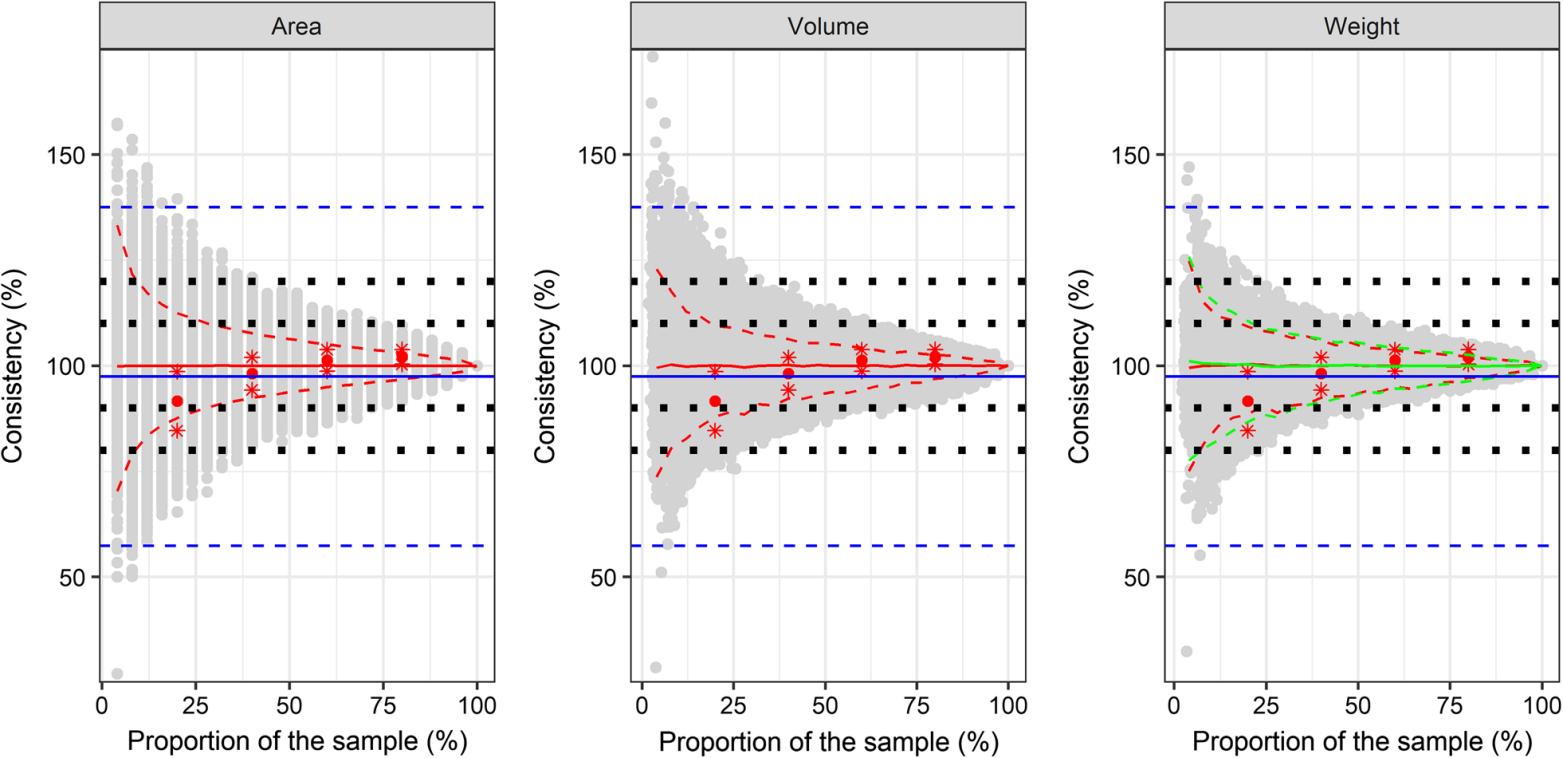
Consistency for the estimated number of specimens calculated by a subsample based on area, volume or weight. Grey points indicate the consistency for the estimated number of specimens for the bootstrapped subsampling of grid cells. The weight data are based on dry weight, fresh weight data are shown in Additional file 9: Figure S6. The red lines for the dry weight and green lines for the fresh weight indicate the bootstrapped mean (solid) and 95% confidence intervals (dashed) of the subsampling dataset. Red points (mean) and red stars (standard deviation) indicate the results of proportional sampling with 20, 40, 60 and 80% of the grid cells. Blue lines indicate mean (solid) and standard deviation (dashed) of the estimation with the image processing software ImageJ over all mosquito samples. Black squares indicate optical orientation lines for a 10 or 20% error

The consistency of the estimated number of specimens per sample did not differ between the subsampling methods based on area, volume or weight (Fig. 2). The analysis of 20% of the sample resulted in an error rate of approximately 12%. An increase of the analyzed proportion to up to 40% further reduced the error to ~8%, i.e. the consistency between the estimates and the actual values increase. Using the optimal combinations of THRESHOLD (replicate a: 57; b: 53; c: 56) and SIZE (replicate a: 50: b: 63; c: 77) in the picture processing software ImageJ was similar to the consistency achieved by subsampling 15–20% with an area/volume/weight-based approach (Fig. 2). The estimated number of specimens with ImageJ and the actual number of specimens were statistically significantly correlated (replicate a: *r* = 0.84; b: *r* = 0.88; c: *r* = 0.82, *P* < 0.001 for all three replicates).

**Fig. 3:**
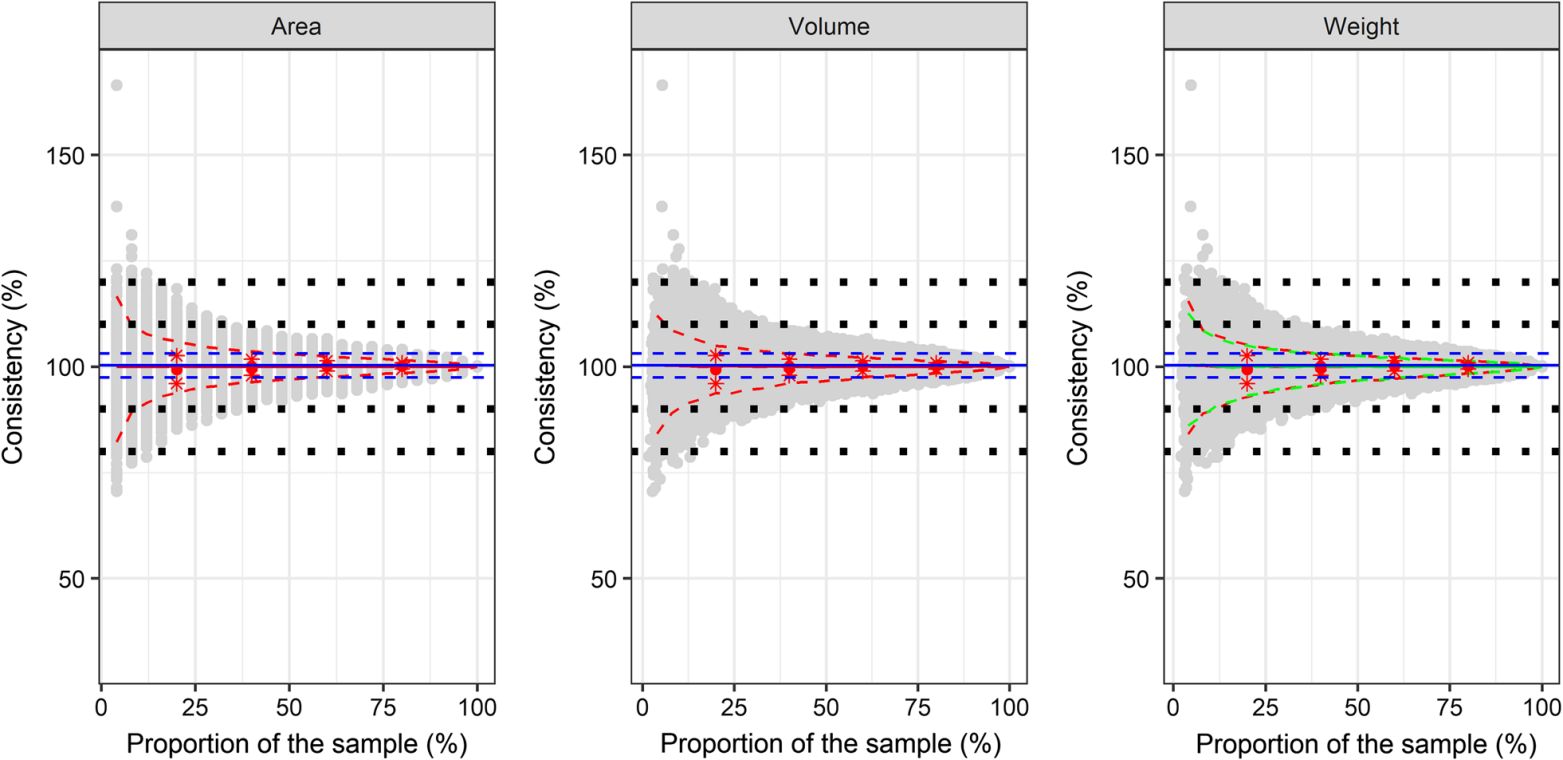
Consistency for the estimated number of specimens for the most abundant species per sample calculated by a subsample based on area, volume or weight. The one most abundant species may vary from sample to sample. The estimated number of specimens for the most abundant species per sample was calculated relative to the actual number of specimens. Grey points indicate the consistency for the estimated number of specimens for the bootstrapped subsampling of grid cells. The weight data are based on dry weight, fresh weight data are shown in Additional file 10: Figure S7. The red lines for the dry weight and green lines for the fresh weight indicate the bootstrapped mean (solid) and 95% confidence intervals (dashed) of the subsampling dataset. Red points (mean) and red stars (standard deviation) indicate the results of proportional sampling with 20, 40, 60 and 80% of the grid cells. Blue lines indicate mean (solid) and standard deviation (dashed) of the estimation with the random subsampling of 200 specimens over all mosquito samples. Black squares indicate optical orientation lines for a 10 or 20% error

For the proportion of the most abundant species per sample, the analysis of 20% of the sample resulted in an error rate of approximately 6% for the number of specimens (Fig. 3). Further increasing the analyzed proportion to 40% reduced the estimation error to ~4%. The random selection of 200 specimens allowed a precise estimation of the proportion of the most abundant species (*r* = 0.97, *P* < 0.001), which corresponds to an analysis of 40% of the total sample (Fig. 3).

**Fig. 4:**
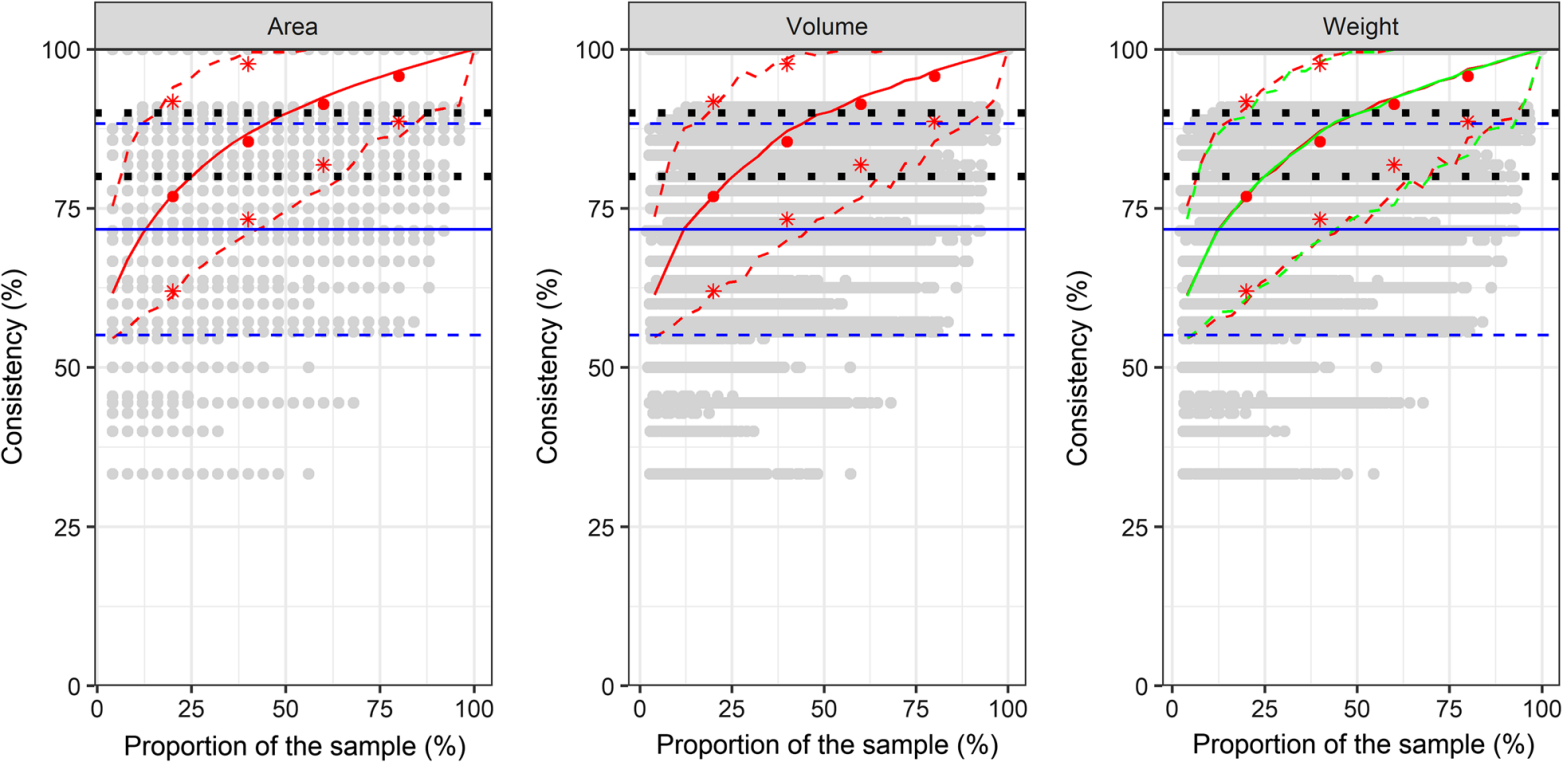
Consistency for the estimated number of species calculated by a subsample based on area, volume or weight. Grey points indicate the consistency for the estimated number of specimens for the bootstrapped subsampling of grid cells. The weight data are based on dry weight, fresh weight data are shown in Additional file 11: Figure S8. The red lines for the dry weight and green lines for the fresh weight indicate the bootstrapped mean (solid) and 95% confidence intervals (dashed) of the subsampling dataset. Red points (mean) and red stars (standard deviation) indicate the results of proportional sampling with 20, 40, 60 and 80% of the grid cells. Blue lines indicate mean (solid) and standard deviation (dashed) of the estimation with the random subsampling of 200 specimens over all mosquito samples. Black squares indicate optical orientation lines for a 10 or 20% error

The real number of species was significantly underestimated regardless of the subsampling method (Fig. 4). As for the estimation of the number of specimens, the accuracy of the methods based on the area, volume and weight did not differ substantially. Sorting 20% of the sample resulted in an average error of 23% (95% CI: 6–40%) for the number of species. Increasing the proportion of the analyzed sample to 40% reduced the error rate for the number of species to 13% (95% CI: 1–30%). Estimation of the number of mosquito species based on the random selection of 200 specimens had a relatively high average number for missing species of 28%, which corresponds to sorting 12% of the total sample (Fig. 4). The correlation between the detected and actual number of species was low (*r* = 0.42, *P* = 0.04).

The selection of adjacent grid cells in comparison to random sampling of grid cells did not alter the accuracy of the estimate. This observation applies to the number of specimens (Fig. 2, Additional file 6: Figure S3), the proportion of the most abundant species (Fig. 3, Additional file 7: Figure S4) and the number of species per sample (Fig. 4, Additional file 8: Figure S5).

The weight of the subsamples after the drying step was significantly lower than before (*t*_(2777)_ = −50.98, *P* ≤ 0.001). Drying reduced the sample weight by an average of 27.3% (standard deviation: 17.8%). However, the estimated number of specimens and species did not differ between dry and fresh weight (Figs. 2, 3, 4, Additional file 9: Figure S6, Additional file 10: Figure S7, Additional file 11: Figure S7).

## Discussion

This study evaluated five different methods to estimate the number of mosquito specimens and species per sample based on four subsampling methods (area, volume, weight and 200 randomly selected individuals) and the image processing software ImageJ. The three approaches based on the area, weight or volume of the subsamples gave very similar results. An analysis of about 20% of the sample resulted in an estimation error of 12% for the actual number of specimens, 6% for the relative abundance of the most abundant species and between 6–40% for the actual number of species. In concordance, Reinert [15] recommended to analyze at least 25% of the specimens with a minimum of 100 specimens to reliably estimate the mosquito abundance and species composition.

One important prerequisite of the area-based subsampling method is an even distribution of the mosquito specimens between all grid cells. Clustered patterns generally cannot be completely avoided [44], e.g. fewer specimens are found in the peripheral cells. Therefore, different subsampling studies recommend a random selection of grid cells to allow a less biased estimation [44–47]. This significantly increases the sample processing time, because the mosquito specimens must be individually picked up. However, the results presented herein reveal that a laborious random selection of grid cells is not necessary to ensure reliable results. Depending on the sample size and the size of the most common species, the size of the grid paper might be adjusted to allow an even distribution of the sample.

Alternative estimation methods are subsampling techniques based on weight or volume. The collection of the sample weight is more laborious compared to the area or volume [35], i.e. weighing of the container, transferring of the sample into the container, recording the weight and re-transferring the sample for further processing. Compared to the wet weight, extrapolations based on dry weight are expected to increase accuracy of the estimation. Drying reduces the variation of the water content between different samples, e.g. caused by differences in the water content of different sized species [48]. However, this processing step causes this method to be even more time consuming [24, 25, 49]. This might explain why most mosquito studies using this method do not mention a drying step [18, 22, 23, 26, 27, 29–32]. Nevertheless, the usage of the dry weight of the subsamples only slightly improved the estimation for the number of specimens and species compared to the use of the fresh weight. Both, dry and wet weight, gave similar results compared to the area-based approach. The same applies when comparing with a volumetric subsampling method. This technique is considered to be only reliable if the majority of the species per subsample have a similar size [16]. Differently-sized species in different frequencies between the subsamples increase the difference between the estimation compared to the actual numbers [46, 50]. Such disadvantage resulting into a lower accuracy of the volumetric approach compared to the other subsampling methods was not observed. This might be explained by relative small size differences between the different mosquito species in our samples.

Using image processing software as an automatized counting tool was highly effective. It only takes marginally longer to prepare the sample for the standardized picture if the total catch is very large, because the effort to remove non-mosquito bycatch or to equally distribute the specimens on the sheet of paper does not change significantly. Nevertheless, this approach is not suitable for differentiating between species, discriminate sexes or feeding status [35, 51]. More research is required to develop algorithms to identify typical characteristics of species, sexes and gonotrophic states, e.g. invasive taxa with a distinct coloration.

The random selection of specimens to estimate the number of mosquito species is commonly found in different mosquito studies selecting between 30–500 specimens [19, 25, 26]. These estimation results are to be interpreted with caution. On average, about 28% of the actual number of species per sample was not recorded with this method for the tested samples in the present study. In contrast, as demonstrated before [18, 19], the random selection of specimens was highly precise to detect the proportion of the most abundant species per sample. A fixed number of random specimens might reduce the comparability between the subsampling results for different mosquito samples. For example, the selection of a fixed number of 200 specimens per sample would result in a high proportion of selected specimens (90%) for a sample with a total of 220 mosquitoes, but less than 10% for samples with more than 2000 mosquitoes. Thus, the appropriate number of randomly selected specimens must depend on the size and the species diversity of the sample. Our results indicate that a huge proportion of the sample must be identified to detect most species. The analysis of at least 25% is required for an average deviation of 20% compared to the actual number of species, which is also supported by Barbour and Gerritsen [45]. Therefore, it is certainly not advisable to subsample by a fixed number of specimens but adapt the size of the subsample to the size of the sample. In addition, in order to minimize the number of non-detected species and to get a more representative estimation of species richness, a visual check of the unsorted part of the sample might be advisable in order to detect rare species [52]. Furthermore, it must be kept in mind, that the representativeness of the sample and subsamples for species richness and species abundance is also affected by biases inherent to the applied trapping methods [53]. Otherwise, the absence of species only found in small numbers can lead to misjudges regarding taxa richness or composition of the vector community.

## Conclusions

Our study demonstrated that the random selection of a fixed number of specimens is by far the fastest method to estimate the proportion of the most abundant species, e.g. to decide whether control activities against nuisance species must be carried out. Nevertheless, this approach only has an insufficient accuracy for a comprehensive analysis of the species composition. This also applies to the sample analysis with the image processing software ImageJ, which can give a good estimation of the number of specimens, but no information on the presence of different species. Therefore, as an operational subsampling strategy, the area-based estimation method of 20% of the sample is probably the method of choice for most kinds of mosquito studies. This approach provided relative precise estimates of the number of specimens (12% error rate) and species per sample (6–40% error rate) and, at the same time, required significantly less effort compared to volume- and weight-based approaches.

## Additional files

**Additional file 1: Table S1.** Number of mosquito specimens and taxa for each sample analysed.

**Additional file 2: Table S2.** Number of mosquito specimens per mosquito taxa for each sample analysed.

**Additional file 3: Figure S1.** Sheet of paper (21.0 × 29.7 cm) used for subsampling subdivided into 25 grid cells (4.2 × 5.9 cm per cell) and 200 blue points.

**Additional file 4: Figure S2.** Adjacent grid cells selected for proportional subsampling (20, 40, 60 or 80%) in a single step.

**Additional file 5: Text S1.** Script to automatically analyse photos in ImageJ.

**Additional file 6: Figure S3.** Consistency for the estimated number of specimens calculated for a proportional subsample (20, 40, 60 and 80%) of the grid cells.

**Additional file 7: Figure S4.** Consistency for the estimated number of specimens for the most abundant species per sample, calculated for a subsample of 20, 40, 60 and 80% of the grid cells.

**Additional file 8: Figure S5.** Consistency for the estimated number of species per sample, calculated for a subsample of 20, 40, 60 and 80% of the grid cells.

**Additional file 9: Figure S6.** Consistency for the estimated number of specimens calculated by a subsample based on fresh weight.

**Additional file 10: Figure S7.** Consistency for the estimated number of specimens for the most abundant species per sample, calculated by a subsample based on fresh weight.

**Additional file 11: Figure S8.** Consistency for the estimated number of species per sample calculated by a subsample based on fresh weight.

## Data Availability

The data supporting the conclusions of this article are included within the article and its Additional files.
