# Supplementary figures and images for: Comparative analysis of subsampling methods for large mosquito samples
Source: Parasit Vectors. 2019 Jul 16;12:354. doi: 10.1186/s13071-019-3606-5 (PMC6636137; doi:10.1186/s13071-019-3606-5)

A 1

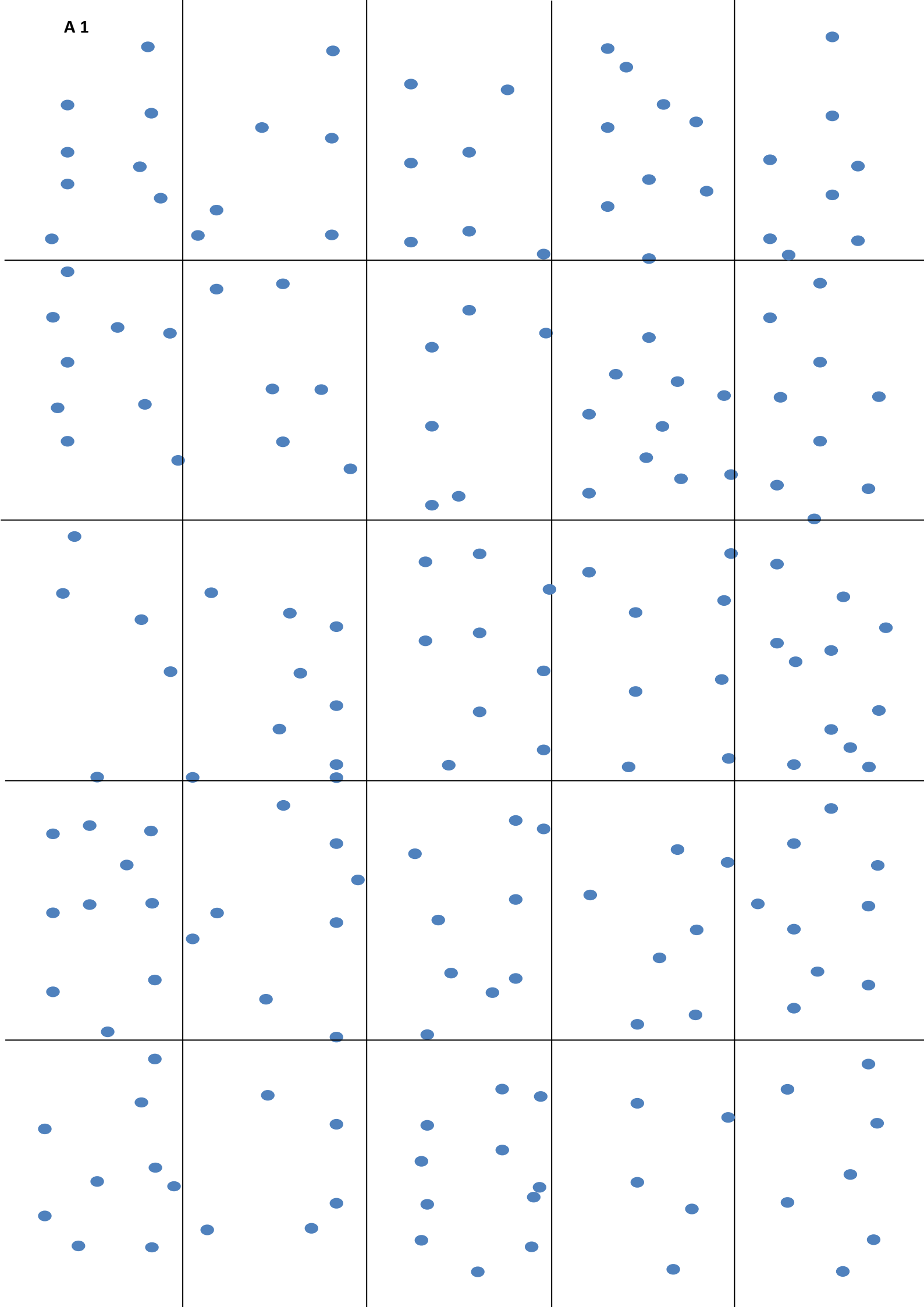

Supplement: Supplementary file 3 — Additional file 3: Figure S1. Sheet of paper (21.0 × 29.7 cm) used for subsampling subdivided into 25 grid cells (4.2 × 5.9 cm per cell) and 200 blue points. [file 13071_2019_3606_MOESM3_ESM.pdf]

20%

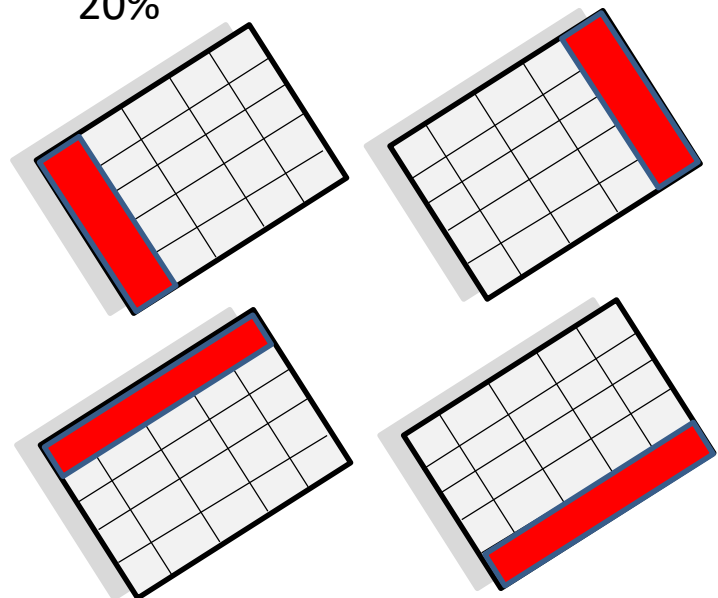

40%

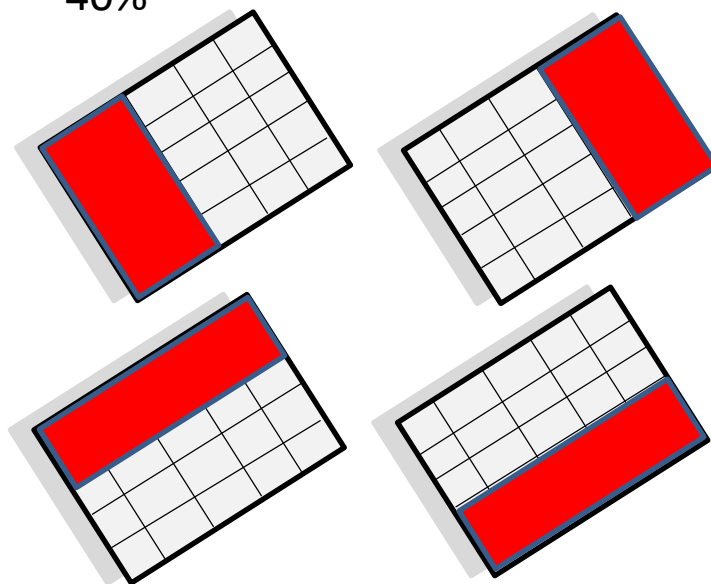

60%

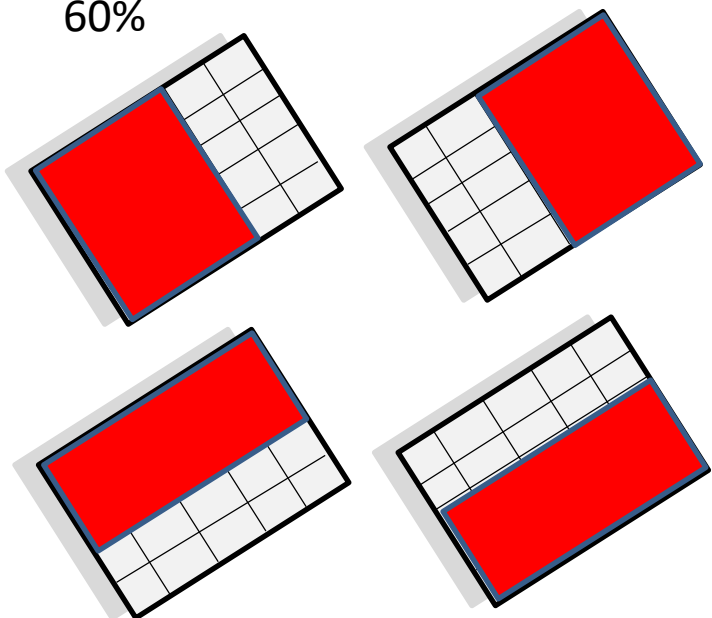

80%

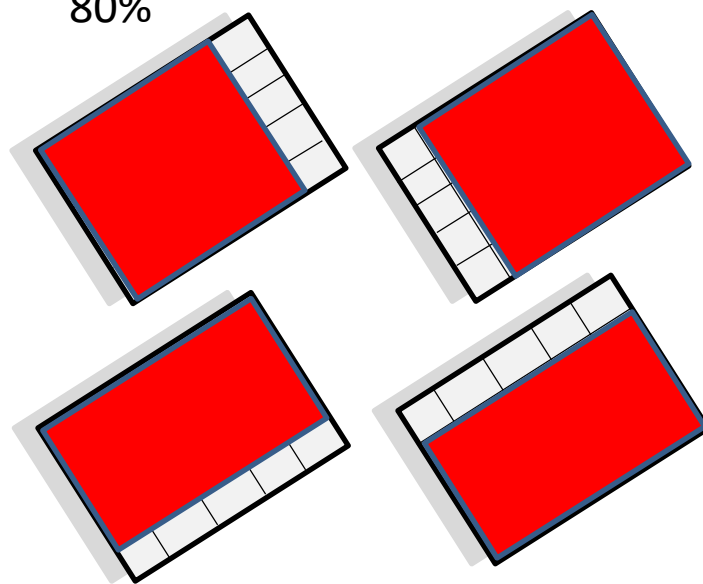

Supplement: Supplementary file 4 — Additional file 4: Figure S2. Adjacent grid cells selected for proportional subsampling (20, 40, 60 or 80%) in a single step. [file 13071_2019_3606_MOESM4_ESM.pdf]
