# Supplementary material for: Comparative analysis of subsampling methods for large mosquito samples
Source: Parasit Vectors. 2019 Jul 16;12:354. doi: 10.1186/s13071-019-3606-5 (PMC6636137; doi:10.1186/s13071-019-3606-5)
Supplement: Supplementary file 6 — Additional file 6: Figure S3. Consistency for the estimated number of specimens calculated for a proportional subsample (20, 40, 60 and 80%) of the grid cells. [file 13071_2019_3606_MOESM6_ESM.pdf]

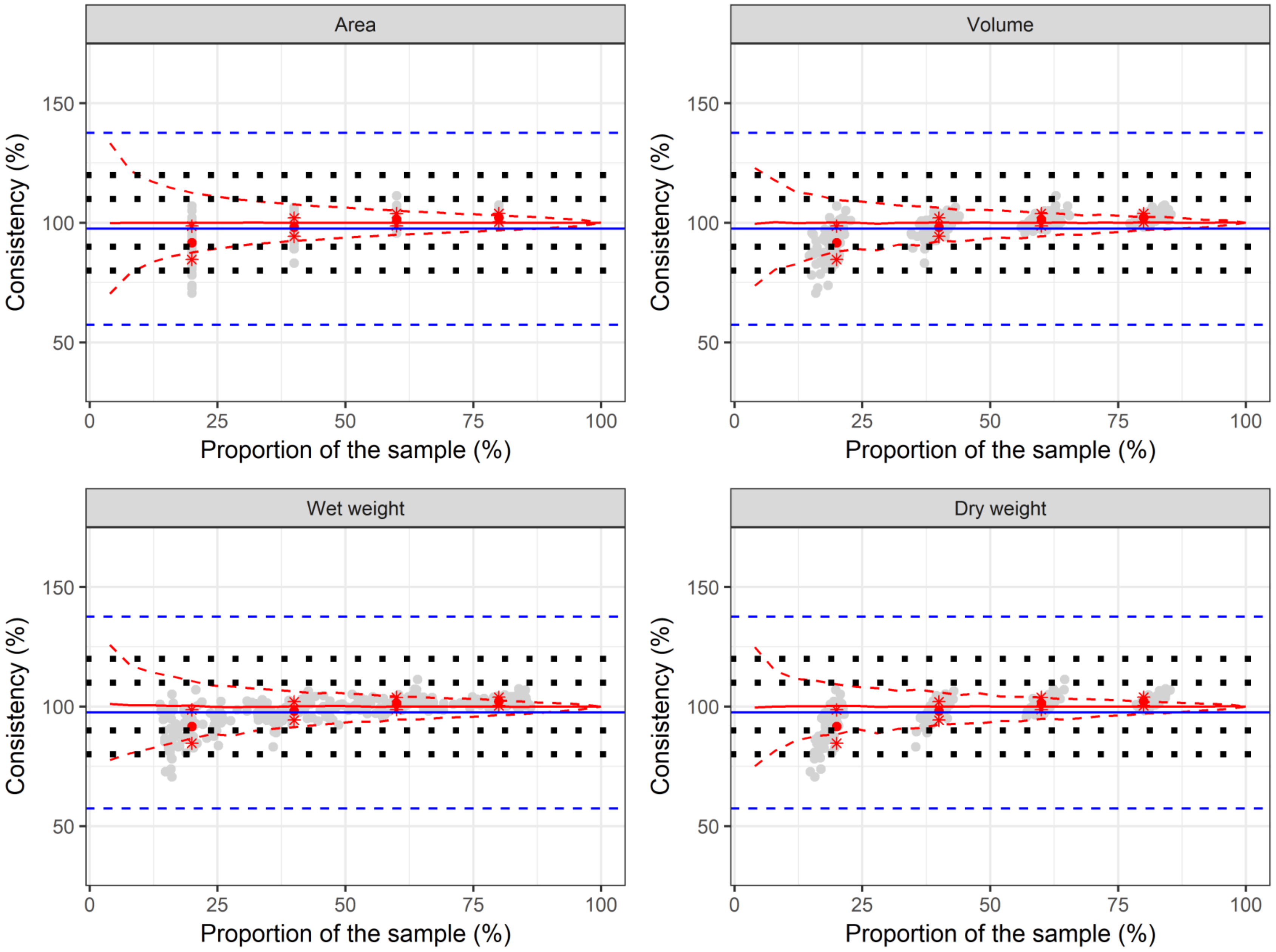

Additional file 6: Figure S3. Consistency for the estimated number of specimens calculated for a proportional subsample (20, 40, 60 and 80%) of the grid cells. Grey points, red points (mean) and red stars (standard deviation) indicate the consistency for the estimated number of specimens of these proportional samples. Red lines indicate the bootstrapped mean (solid) and 95% confidence intervals (dashed) of the bootstrapped subsampling dataset. Blue lines indicate mean (solid) and standard deviation (dashed) of the estimation with the image processing software ImageJ. Black squares indicate the 10 and 20% error
