# Supplementary material for: Comparative analysis of subsampling methods for large mosquito samples
Source: Parasit Vectors. 2019 Jul 16;12:354. doi: 10.1186/s13071-019-3606-5 (PMC6636137; doi:10.1186/s13071-019-3606-5)
Supplement: Supplementary file 9 — Additional file 9: Figure S6. Consistency for the estimated number of specimens calculated by a subsample based on fresh weight. [file 13071_2019_3606_MOESM9_ESM.pdf]

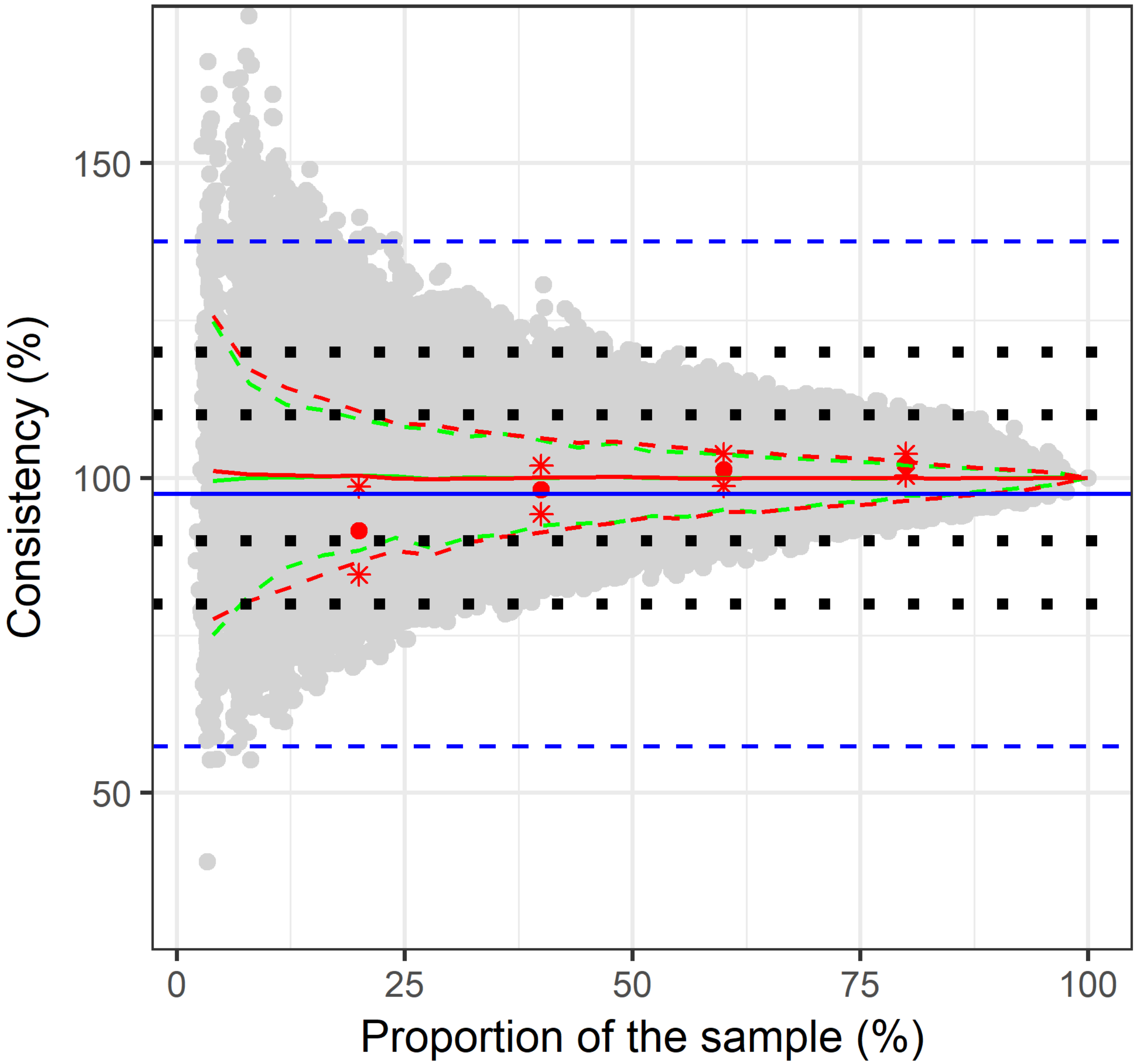

Additional file 9: Figure S6. Consistency for the estimated number of specimens calculated by a subsample based on fresh weight. Grey points indicate the consistency for the estimated number of specimens of the bootstrapped subsampling of grid cells. The red lines for the fresh weight and green lines for the dry weight indicate the bootstrapped mean (solid) and 95% confidence intervals (dashed) of the subsampling dataset. Red points (mean) and red stars (standard deviation) indicate the results of proportional sampling with 20, 40, 60 and 80% of the grid cells. Blue lines indicate mean (solid) and standard deviation (dashed) of the estimation with the image processing software ImageJ. Black squares indicate 10 and 20% error
